# Promoter methylation of *P16*, *RARβ*, E-cadherin, cyclin A1 and cytoglobin in oral cancer: quantitative evaluation using pyrosequencing

**DOI:** 10.1038/sj.bjc.6602972

**Published:** 2006-01-31

**Authors:** R J Shaw, T Liloglou, S N Rogers, J S Brown, E D Vaughan, D Lowe, J K Field, J M Risk

**Affiliations:** 1 Molecular Genetics & Oncology Group, School of Dental Sciences, University of Liverpool, Liverpool L69 3GN, UK; 2 University of Liverpool Cancer Research Centre, Roy Castle Lung Cancer Research Programme, 200 London Rd, Liverpool L3 9TA, UK; 3 Regional Maxillofacial Unit, University Hospital Aintree, Longmoor Lane, Liverpool L9 7AL, UK

**Keywords:** methylation, oral squamous cell carcinoma, pyrosequencing

## Abstract

Methylation profiling of cancer tissues has identified this mechanism as an important component of carcinogenesis. Epigenetic silencing of tumour suppressor genes through promoter methylation has been investigated by a variety of means, the most recent of which is pyrosequencing. We have investigated quantitative methylation status in oral squamous cell carcinoma patients. Fresh tumour tissue and normal control tissue from resection margin was obtained from 79 consecutive patients undergoing resection of oral squamous cell carcinoma. DNA was extracted and bisulphite treated. PCR primers were designed to amplify 75–200 bp regions of the CpG rich gene promoters of *p16*, RAR*β*, E-cadherin, cytoglobin and cyclinA1. Methylation status of 4-5 CpG sites per gene was determined by pyrosequencing. Significant CpG methylation of gene promoters within tumour specimens was found in 28% for *p16*, 73% for *RARβ*, 42% for E-cadherin, 65% for cytoglobin and 53% for cyclinA1. Promoter methylation was significantly elevated in tumours compared to normal tissue for p16 (*P*=0.048), cytoglobin (*P*=0.002) and cyclin A1 (*P*=0.001) but not in *RARβ* (*P*=0.088) or E-cadherin (*P*=0.347). Concordant methylation was demonstrated in this tumour series (*P*=0.03). Significant differences in degree of methylation of individual CpG sites were noted for all genes except *RARβ* and these differences were in a characteristic pattern that was reproduced between tumour samples. Cyclin A1 promoter methylation showed an inverse trend with histological grade. Promoter methylation analysis using pyrosequencing reveals valuable quantitative data from several CpG sites. In contrast to qualitative data generated from methylation specific PCR, our data demonstrated *p16* promoter methylation in a highly tumour specific pattern. Significant tumour specific methylation of cyclin A1 promoter was also seen. Cytoglobin is a novel candidate tumour suppressor gene highly methylated in upper aero-digestive tract squamous cancer.

The role of promoter hypermethylation has become a focus for research in many tumour sites, including head and neck cancer (HNSCC) (Fan, 2004). Silencing of certain tumour suppressor genes, central to the development of many solid tumours, may occur in the absence of genetic change, via aberrant methylation of CpG islands (Esteller, 2003). Several promising avenues exist in attempting to translate this research field into the clinical management of oral squamous cell cancer (OSCC). Attempts to monitor and prognosticate on the malignant transformation of oral dysplasia using methylation status have been reported (Kresty *et al*, 2002; McGregor *et al*, 2002; Yeh *et al*, 2002; Lopez *et al*, 2003). Early diagnosis, molecular staging and tumour surveillance of OSCC have also been explored, with particular emphasis on minimally invasive methods such as oral rinsing or blood testing (Sanchez-Cespedes *et al*, 2000; Ogi *et al*, 2002). Furthermore, epigenetic changes, at least in principal, are reversible by pharmacological means and thus offer potential therapeutic targets (Coombes *et al*, 2003).

Published studies have identified a methylation profile or ‘methylotype’ of OSCC by the study of a limited number of genes. In particular, methylation of the promoters of *P14*, *P15*, *P16*, *RASSF1*, *MGMT*, DAP Kinase, and Sigma 14-3-3 have been well described (Shaw, 2006). Some genes have been selected by their potential correlation with clinical behaviour for example, the role of E-cadherin in cell/cell adhesion and metastasis (Kudo *et al*, 2004) and of *RARβ* expression in the prediction of response to chemo-preventative agents (Wan *et al*, 1999). Efforts have been made to discover genes where promoter methylation is specific to, and present in a high percentage of cases of, malignant tissue and is not found in normal oral mucosa. These requirements demand well-controlled studies and, ideally, quantitative methods of methylation analysis. Microarray technology and pharmacological reversal of promoter methylation has lead to the identification of new candidate genes thought to be epigenetically silenced in HNSCC (Tokumaru *et al*, 2004), but the involvement of these genes requires individual verification.

Several methods of determination of promoter methylation have been described including the use of restriction enzymes (Singer-Sam *et al*, 1990), genomic bisulphite sequencing (Frommer *et al*, 1992) and microarray-based methylation analysis (Huang *et al*, 1999), however, the overwhelming majority of published data uses methylation-specific PCR following bisulphite treatment (MSP) (Herman *et al*, 1996). MSP has been successfully performed in a large number of studies providing useful results, mainly due to its increased sensitivity. However, it yields qualitative rather than quantitative data and usually evaluates only a few CpG sites at the 3′ end of the primers. It also lacks internal control for adequacy of bisulphite treatment (all non-5′-methylated cytosines should be fully converted to thymine), making identification of false positives very difficult. MSP is also potentially liable to oversensitivity following the high number of PCR cycles sometimes reported, for example 80 cycles (An *et al*, 2002). While several methods have been developed to overcome these shortcomings, pyrosequencing offers a semiquantitative, high throughput and reliable method with inbuilt internal control for adequacy of bisulphite treatment (Colella *et al*, 2003; Tost *et al*, 2003; Dupont *et al*, 2004).

We have evaluated a consecutive series of previously untreated primary oral cancers using pyrosequencing to quantify promoter methylation in five genes. The commonly investigated genes, *P16, RARβ* and E-cadherin (*ECAD*) were included in this study to allow comparison with the published MSP literature. We also investigated the promoters of the cyclin A1 (*CYCA1*) gene, which has recently been suggested as a promising candidate gene for epigenetic silencing in HNSCC (Tokumaru *et al*, 2004), and the cytoblogin (*CYGB*) gene, which has been implicated in oesophageal and lung cancer within our own group (unpublished), but not previously investigated in OSCC. Quantitative data is presented in the context of normal controls and also in the light of correlation with detailed clinical and histopathological data.

## MATERIALS AND METHODS

### Patients

A total of 79 consecutive patients presenting to the Regional Maxillofacial Unit, University Hospital Aintree, Liverpool, UK (www.headandneckcancer.co.uk) with previously untreated squamous cell carcinoma of the oral cavity or oro-pharynx were consented for tissue collection following appropriate ethical committee approval. Only those patients undergoing surgery as the primary treatment were recruited and we estimate that this includes approximately 90% of all presentations. Demographic, clinical, pathological and outcome characteristics of each patient were recorded within the unit's dedicated head and neck database (Rogers *et al*, 1996). Detailed histology data was recorded (Helliwell and Woolgar, 2005), providing histopathological grading and staging.

### Clinical and pathological characteristics

In all, 52 of the 79 patients were male (66%) with an age range 29–91 years (median 60 years). In all, 45 (57%) were heavy smokers (>20 pack years) and 39 (49%) admitted to alcohol use >28 U/week. The commonest sites of tumours were anterior floor of mouth 20 (25%), oral tongue 18 (23%), tonsil nine (11%) and maxilla eight (10%). Histopathological assessment following surgical resection staged the cases as follows: pT1: 14 (18%); pT2: 30 (38%); pT3: 9 (11%); pT4: 26 (33%). In all, 32 cases (41%) were pN positive and 21 (27%) had extra-capsular spread.

### Tissue collection

Tumour samples 5 mm^3^ were excised from resected specimens within the tumour mass, but not involving the margin. Normal samples of similar dimension were taken from the resection margin, approximately 10 mm from the macroscopic tumour edge and subsequently confirmed as benign by routine histopathology. A total of 80 tumour and 25 normal tissue samples were collected (one patient had synchronous primary tumours), snap frozen in liquid N_2_ and subsequently stored at −85°C.

### DNA extraction and bisulphite treatment

DNA was extracted from 2 mm^3^ tissue samples using a DNeasy™ tissue kit (Qiagen Ltd). DNA concentration was measured by spectrophotometry and subsequently adjusted to 40 ng/*μ*l. Bisulphite treatment of 2 *μ*g of each sample was undertaken using the EZ DNA Methylation Kit™ (Zymo Research) and the converted DNA eluted in 50 *μ*l of 0.1 × TE buffer. Human genomic DNA (4 *μ*g) was artificially methylated (Matsuo *et al*, 1994) as a positive control using *Sss*I (CpG) Methylase (New England Biolabs®).

### Promoter CpG island analysis and primer design

**Figure 1:**
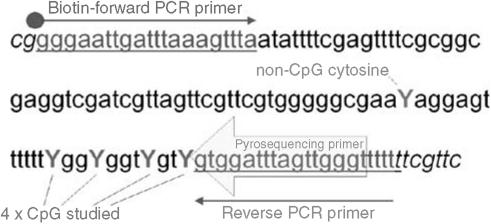
PCR and sequencing primers for the cytoglobin gene promoter.

PCR assays were designed to amplify a part of the CpG islands in the examined gene promoters. Primers targeted CpG-free regions to ensure that the PCR product would proportionally represent the methylation characteristics of the source DNA. Pyrosequencing primers were subsequently designed to focus on a series of four or five ‘target’ CpG dinucleotides and again avoided CpGs within the primer sequence (Figure 1). The targeted CpGs were chosen, where possible (P16, RAR*β*, E-cad) to correlate with those specified by standard MSP primers previously described. In the case of Cyclin A1 and CYGB, target regions were selected in line with the demands of primer design as described.These target CpGs were evaluated by analysis of the resulting pyrogram. Guanine (or cytosine if a forward sequencing primer is used) is incorporated during pyrosequencing if the template CpG was methylated, while adenine (or thymine) is incorporated if the template CpG was unmethylated. Thus the proportion of G : A (or C : T) incorporated is stoichiometrically proportional of the degree of methylation at that CpG site in the template DNA. The analysis of a non-CpG cytosine provides an internal control of the completeness of bisulphite treatment.

### Pyrosequencing methylation analysis

Hot-start PCR was carried out with HotStar Taq® Master Mix Kit (Qiagen Ltd.) using 3 *μ*l bisulphite treated DNA. Confirmation of PCR product quality and freedom from contamination was established on 2% agarose gels with ethidium bromide staining. Pyrosequencing was carried out using the PSQ96MA System (Biotage) according to manufacturer's protocol, including single strand binding protein (PyroGold reagents). PCR primer sequences, PCR conditions and sequencing primer sequences are given in Table 1.

### Data analysis

The methylation index (MtI) at each gene promoter, and for each sample, was calculated as the average value of ^m^*C*/(^m^*C*+*C*) for all examined CpGs in the gene.

Statistical correlations between MtI and the clinical variables recorded were made using SPSS version 11.

## RESULTS

### Methylation indices

**Figure 2:**
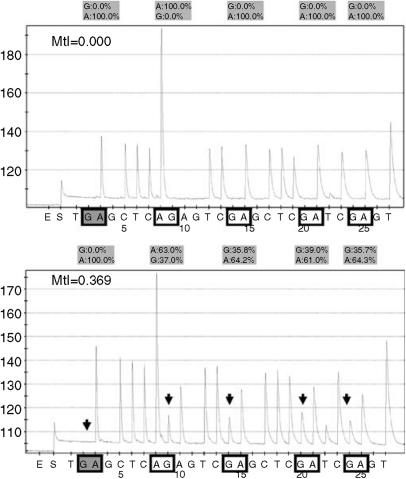
Representative pyrograms for *RARβ.* The four targeted cytosines are enclosed in unshaded squares (as reverse strand was read, G peaks (arrowed) indicate methylated cytosine while (A) indicates unmethylated cytosine). The control, non-CpG cytosine residue showing complete conversion of cytosine to uracil by bisulphite treatment is shown in the left-hand shaded box. Normal tissue (top panel) demonstrates no methylation while tumour tissue (bottom panel) demonstrates a significant level of methylation at all four target bases. The Methylation Index (MtI) is calculated as the average rate of G incorporation at each CpG.

**Figure 3:**
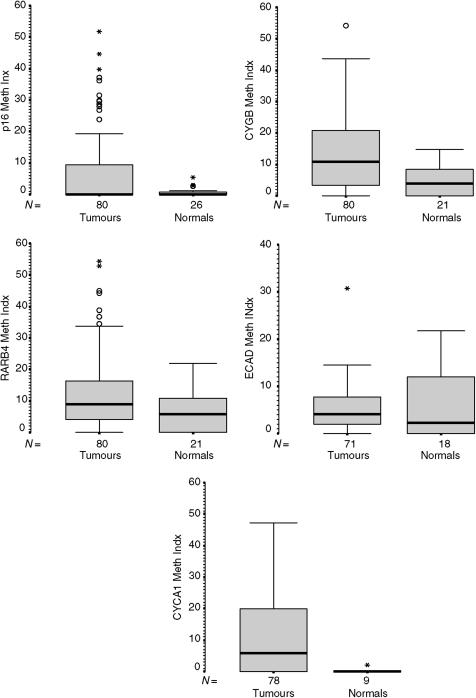
Box and whisker plots of methylation Indices for the five genes studied. Boxes include 50% data, O=Outlier ‘cases with values between 1.5 and 3 box lengths from the upper or lower edge of the box. The box length is the interquartile range.’ ^*^=Extreme ‘cases with values more than three box lengths from the upper or lower edge of the box’.

Example bisulphite methylation profiles are shown in Figure 2. The distribution of methylation indices (MtI) for tumours and normals is presented in Table 2. There were no tumour samples that had a zero methylation index for all five genes, however, low-level methylation (0–5%) was relatively common and we consider this to represent background ‘noise’ with questionable significance. The mean MtIs for the positive control DNA (artificially methylated) were *P16*: 0.722; *RARβ*: 0.165; *CYGB*: 0.849; *CYCA1*: 0.812; *ECAD*: 0.702. Tumour MtI was significantly higher than that of normal tissue in the case of *P16*, *CYGB* and *CYCA1* but not *RARβ* or *ECAD* (Kruskall–Wallis test of mean rank) (Table 2). This contrast is illustrated by the box and whisker plots of *P16* and *ECAD* MtI distribution in Figure 3. A total of 74 of the tumour samples (95%) demonstrated significant methylation (MtI>0.05) at one or more gene promoter. Pyrosequencing data was successfully generated in 100% of tested samples for *P16*, 95% for *RARβ*, 95% for *CYGB*, 84% for *ECAD* and 82% for *CYCA1*. The internal controls to check for adequacy of bisulphite treatment, that is methylation of non-CpG cytosines, suggested 100% of the DNA samples used were satisfactory in this regard. Samples with failed results were repeated, where possible by repreparing DNA from the original tumour specimens, however, this process added little to the results suggesting that deletions may have been present in the region of the gene promoter.

### Correlation between clinico-pathological and methylation data

There was little evidence of significant correlation between MtI and the clinical, demographic or pathological data studied. However, methylation at the *CYCA1* gene promoter showed a trend for lower Anneroth score (Anneroth *et al*, 1986), a histological grading system (Spearman's rank: *ρ*=−0.253, *P*=0.05, but multiple correlations made this NS). The significance of this is unclear as no corresponding correlations were found with tumour thickness, lymph node involvement or margin status, any or all of which might reasonably have been expected to correlate with histological grade.

Significant correlations, however, were seen between methylation at *RARβ* and both *P16* (*ρ*=0.29, *P*=0.01 Spearman's rank) and *CYGB* (*ρ*=0.45, *P*<0.001). Weaker correlations were seen between methylation at *ECAD* and *CYCA1* (*ρ*=0.24, *P*=0.04)*, ECAD* and *CYGB (ρ*=−0.22, *P*=0.07), and between *P16* and *CYGB* (*ρ*=0.19, *P*=0.10). This apparent interdependence of promoter methylation of genes, recently described as ‘concordant methylation’ (An *et al*, 2005), was subsequently confirmed by goodness of fit testing (*χ*^2^:12.8 with 5 df, *P*=0.03 using methylation rates >5% for each gene to derive the expected number of genes with methylation if independent). The number of cases with significant methylation (MtI>0.05) at four or five of the genes studied was higher than would be expected (by a ratio of 2.1 : 1) if methylation was an unrelated or random event.

**Figure 4:**
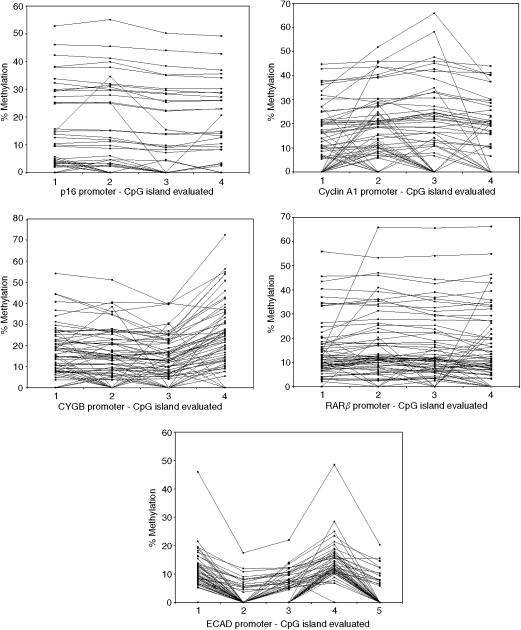
CpG methylation patterns within the promoter regions of the five genes studied.

For each gene, the percentage of methylation at each individual CpG varied in a characteristic pattern that was reproduced between tumour samples. This is best illustrated by *ECAD* promoter methylation (Figure 4). Similar but less exaggerated patterns were also seen in the other genes and, in all except *RARβ*, the differences in methylation of different CpGs were significant (Friedman's test: *P16*, *ECAD*, *CYGB*, *CYCA1*: *P*<0.001; *RARβ P*=0.129). These patterns of methylation were also observed in the positive control DNA prepared using DNA methylase.

## DISCUSSION

In this study, we have used pyrosequencing to determine the methylation profile of oral squamous cell carcinoma in a semi-quantitative manner. Our data demonstrates tumour-specific promoter methylation is found in a significant proportion of cases at *P16, CYGB* and *CYCA1*, while methylation of the promoters of *ECAD* and *RARβ* was also seen in surrounding normal tissues. Patterns of concordant methylation have also been demonstrated.

p16 (INK4a) inhibits G1 to S phase passage by binding cyclin dependent kinase and preventing formation of its complex with cyclin D. Promoter methylation at *P16* has been widely investigated and reported in the literature. Rates of methylation of this promoter in OSCC vary between 31% (Maruya *et al*, 2004) and 67% (Kulkarni and Saranath, 2004). Much of this MSP based literature has also found high levels in surrounding normal tissue (50%) (Kulkarni and Saranath, 2004), dysplastic tissue (33%) (Maruya *et al*, 2004) and leukoplakia 44% (Lopez *et al*, 2003) leading to *P16* methylation being previously described as an early change in oral carcinogenesis of little prognostic value. Using pyrosequencing, we found that significant methylation was found in 28% of tumours but in only 4% of the normal tissues. Our ‘normal’ specimens were taken from the surgical resection margin and whilst frankly involved mucosal margins are uncommon in this institution (Woolgar and Triantafyllou, 2005), there would undoubtedly be a significant rate of dysplasia and molecular field change within these tissues. Drinking and smoking habits, prevalent in this population, might also be expected to induce widespread field change in the upper aero-digestive tract (El Naggar *et al*, 1995; Scully *et al*, 2000) Our finding that *P16* promoter methylation was highly tumour specific contrasts with previous published work using MSP and leads us to consider the benefits of a quantitative rather than qualitative methylation assay. Pyrosequencing analysis of biopsy material from non-cancer patients and also from dysplastic keratosis would clearly be of interest.

Interest in *RARβ* promoter methylation has developed from studies investigating the potential chemo-preventative role of retinoids in OSCC (McGregor *et al*, 2002; Youssef *et al*, 2004a). The effects of retinoids are though to be mediated via nuclear receptors including RAR*β* (Wan *et al*, 1999). MSP based studies have found 67% of tumours to show methylation of this gene promoter (Youssef *et al*, 2004b), and for this methylation to be significantly higher than normal surrounding tissues. Our pyrosequencing data shows significant methylation (MtI>0.05) in 73% of tumour samples, however, the trend for higher MtI in tumour than normal specimens did not reach significance (*P*=0.088). In our data, 62% of the normal specimens had MtI>0.05 and this appears to support existing theories that promoter methylation of *RARβ* is widespread in apparently normal tissues as well as tumour and is, perhaps, an early event in carcinogenesis.

Epigenetic silencing of *ECAD* has been investigated in several studies and rates of approximately 50% in OSCC are reported (Chang *et al*, 2002; Hasegawa *et al*, 2002; Yeh *et al*, 2002; Viswanathan *et al*, 2003; Maruya *et al*, 2004). E-cadherin is a transmembrane glycoprotein responsible for cell–cell adhesion, the reduced expression of which has been correlated with regional metastasis in OSCC. While some studies have found a correlation with promoter methylation and propensity to metastasise (Chang *et al*, 2002), this finding is not undisputed (Yeh *et al*, 2002) and the relationship between promoter methylation and expression appears less clear than in other tumour suppressor genes. Our data suggests that *ECAD* promoter methylation is the least tumour specific of the five genes under investigation (*P*=0.347). An MtI>0.05 was present in 33% of normal tissues and indeed some of the highest MtIs for this gene were seen in normal tissues. Notably, no correlation was found between *ECAD* MtI and metastatic potential as determined by pN stage, number of involved nodes or extra-capsular spread. This contrasts with previous data implicating this protein in metastasis.

Cyclin A1 is a tissue-specifically expressed gene which is strongly methylated in solid tumours (Muller-Tidow *et al*, 2001). A recent study using demethylation unmasking of potentially epigenetically silenced genes in HNSCC found that the *CYCA1* promoter was methylated in 45% of tumours but in none of the normal tissues (Tokumaru *et al*, 2004). Cyclin A1 is involved in apoptosis and growth arrest downstream of p53; interestingly the above study found an inverse correlation between *CYCA1* promoter methylation and the presence of *P53* mutations. The pyrosequencing data in our study also found methylation status to be highly tumour specific (*P*=0.001) with 53% of tumours having an MtI>0.05. Interestingly our data showed a significant inverse trend between *CYCA1* MtI and Anneroth score, however, this was in the absence of significant correlations with individual measures of histological grade and the statistical significance was borderline. *P53* mutations are present in around 50% of OSCC tumours and have been shown in some studies to correlate with poor prognosis and radio-resistance (Schliephake, 2003; Yamazaki *et al*, 2003). It will be interesting to obtain outcome data for our cohort and determine whether those with *CYCA1* Promoter methylation have better prognosis.

*CYGB* is a candidate tumour-suppressor gene on chromosome 17q and is the only gene completely contained within the 42.5 kb Tylosis with Oesophageal Cancer minimal region (Risk *et al*, 2002). This region is subject to frequent deletions in sporadic oesophageal cancer, however, no coding mutations have been demonstrated in affected tylosis patients or a series of squamous oesophageal tumours (Langan *et al*, 2004; Shahabi *et al*, 2004). Cytoglobin is a recently described, intracellular globin (Burmester *et al*, 2002; Burmester *et al*, 2004) whose role in cancer is as yet unclear but may be related to detoxification of oxygen free radicals (Trent, III and Hargrove, 2002). Promoter methylation of *CYGB* in lung cancer has recently become the focus of investigation within our research group and may be implicated in ovarian cancer (Presneau *et al*, 2005). The *CYGB* promoter was found to be significantly methylated in 46% of lung tumour specimens and significantly fewer normal controls. Further the MtI of *CYGB* in lung tumours correlated with RNA expression (*P*=0.001) (unpublished). In OSCC, we now find that the *CYGB* promoter is significantly methylated in 65% of tumours and, further, that methylation is highly tumour specific (*P*=0.002). Recently presented pilot micro-array data also reveals that CYGB is consistently downregulated in head and neck cancer (Thurlow *et al*, 2005). The epigenetic data presented in this study adds weight to the hypothesis that *CYGB* is a tumour suppressor gene highly methylated in upper aero-digestive tract squamous cancer.

The pattern of variable CpG methylation seen within gene promoters is a new finding made possible by the highly detailed data generated from pyrosequencing. Explanations underlying these methylation patterns, as seen in Figure 4, are currently a matter for speculation. It is possible that these changes result from experimental artefact, but there is an observable trend towards lower methylation at any CpG located only a few nucleotides away from a CpG with high methylation. It may be that steric hindrance of DNA methylase does not allow equally high methylation of two closely adjacent CpGs (personal communication: Dr Jorg Tost, Centre National de Genotypage, Evry, France and Professor Ralph Krahe, MD Anderson Cancer Centre, TX, USA). Full explanation of these apparent subtleties, however, awaits further investigation. Our finding is that these patterns of CpG methylation are characteristic in any one gene promoter and highly reproducible between samples. We believe this adds weight to our use of methylation index in analysis, rather than concentrating on an individual single CpG site.

The inter-relationship between promoter methylation at several genes (or ‘concordant methylation’) is worthy of further discussion. The number of cases with significant methylation (MtI>0.05) at four or five of the genes studied was higher than would be expected (21 *vs* 10) if methylation was a random event. This finding suggests the possibility that there is a subgroup of tumours that may have a predominant epigenetic pathogenesis. Other subgroups may have principally genetic pathogenesis with deletions and mutations being more prevalent than promoter methylation. This hypothesis would need to be tested by examining a wide range of both genetic and epigenetic aberrations within the same cohort. The pathological significance of this CpG island methylation phenotype (CIMP) (Issa, 2004) recently described in other tumour sites was not seen in this series. This aspect, however, may be worthy of further investigation when clinical outcome data from our series become available and as further genes are studied. We feel that the quantitative data generated as we describe will add valuable insight to explore this concept.

In conclusion, we have used pyrosequencing technology to uncover further details of the epigenetic profile of oral squamous cell cancer. Our data indicate that *P16* promoter methylation is highly tumour specific, in contrast to some previously published MSP data, whilst *CYCA1* promoter methylation has not previously been investigated in an oral cancer series and also appears to be tumour specific. The role of *CYGB* as a tumour suppressor gene in oral cancer is also novel and awaits further investigation.

## Figures and Tables

**Table 1:** PCR Primers and conditions

**Gene**	**Assay CpG^a^**	**Control C**	**Primers^b^**		**PCR Conditions**			
*p16*	−53		Forward PCR	AGGGGTTGGTTGGTTATTAG		94°C 30 s		
	−57	−51	Reverse PCR	Biotin-CTACCTACTCTCCCCCTCTC	94°C (14:30 m)	58°C 40 s	× 40 cycles	72°C (10:00 m)
	−59		Sequencing (F)	GGTTGGTTATTAGAGGGT		72°C 30 s		
	−64							
	−136							
E-Cadherin (*CDH1*)	−144		Forward PCR	TTTGATTTTAGGTTTTAGTGAGT		94°C 30 s		
	−150	−163	Reverse PCR	Biotin-ACCACAACCAATCAACAA	95°C (14:30 m)	55°C 30 s	× 40 cycles	72°C (10:00 m)
	−158		Sequencing (F)	TAGTAATTTTAGGTTAGAGG		72°C 30 s		
	−160							
								
*RARβ* c	33		Forward PCR	Biotin-GTTAAAGGGGGGATTAGAAT		94°C 30 s		
	35	48	Reverse PCR	CTCCTTCCAAATAAATACTTACAA	94°C (14:30 m)	58°C 40 s	× 40 cycles	72°C (10:00 m)
	39		Sequencing (R)	ACCCAAACAAACCCT		72°C 30 s		
	44							
								
CyclinA1	−589		Forward PCR	Biotin-GAGTTAGGGTTTTTAGGA		94°C 30 s		
	−598	−596	Reverse PCR	CCTCCAACTCCAACTATAC	95°C (14:30 m)	55°C 30 s	× 40 cycles	72°C (10:00 m)
	−600		Sequencing (R)	CTAACAACCCCCTCTA		72°C 30 s		
	−604							
								
Cytoglobin (*CYGB*)	−504		Forward PCR	Biotin-GGGAATTGATTTAAAGTTTA		94°C 30 s		
	−507	−492	Reverse PCR	AAAAAACCCAACTAAATCCAC	95°C (14:30 m)	52°C 45 s	× 40 cycles	72°C (10:00 m)
	−510		Sequencing (R)	ACCCAACTAAATCCAC		72°C 30 s		
	−514							

a Relative to transcription start of gene studied.

b (F): sequencing primer extends in the F direction; (R): sequencing primer extends in the R direction.

c Examined CpGs in 5′UTR.

**Table 2:** Methylation data and analysis

**Gene**	**Samples**	**% samples MtI>0^a^**	**% samples MtI>5%^b^**	**Kruskall–Wallis test of mean rank (diff. T/N)**	**Reference MtI^c^**	**% tumours ⩾ Reference MtI**
*P16*	T	45% (36/80)	28% (22/80)	*P*=0.048	0.046	22/80 28%
	N	27% (7/26)	4% (1/26)			
*RARβ*	T	85% (68/80)	73% (58/80)	*P*=0.088 (NS)	0.215	15/80 19%
	N	71% (15/21)	62% (13/21)			
*CYGB*	T	74% (59/80)	65% (52/80)	*P*=0.002	0.149	35/80 44%
	N	57% (12/21)	52% (11/21)			
*CYCA1*	T	72% (56/78)	53% (42/78)	*P*=0.001	0.002	38/78 49%
	N	11% (1/9)	0% (0/9)			
*ECAD*	T	83% (59/71)	42% (30/71)	*P*=0.347 (NS)	0.218	1/71 1%
	N	56% (10/18)	33% (6/18)			

a MtI=Methylation index (mean of % methylation at all CpGs/100).

b 0–5% methylation is considered to be due to experimental background.

c MtI below which 95% of normal sample data fall.
